# *In vitro* Evaluation of the Colistin-Carbapenem Combination in Clinical Isolates of *A. baumannii* Using the Checkerboard, Etest, and Time-Kill Curve Techniques

**DOI:** 10.3389/fcimb.2017.00209

**Published:** 2017-05-24

**Authors:** Micheline A. H. Soudeiha, Elias A. Dahdouh, Eid Azar, Dolla K. Sarkis, Ziad Daoud

**Affiliations:** ^1^Rodolphe Merieux Laboratory, School of Pharmacy, Saint-Joseph UniversityBeirut, Lebanon; ^2^Animal Health Laboratory, Faculty of Veterinary, Universidad Complutense de MadridMadrid, Spain; ^3^Clinical Microbiology, Faculty of Medicine and Medical Sciences, University of BalamandKoura, Lebanon

**Keywords:** *Acinetobacter* spp, Checkerboard, time kill curve, perpendicular Etest, International clone

## Abstract

The worldwide increase in the emergence of carbapenem resistant *Acinetobacter baumannii* (CRAB) calls for the investigation into alternative approaches for treatment. This study aims to evaluate colistin-carbapenem combinations against *Acinetobacter* spp., in order to potentially reduce the need for high concentrations of antibiotics in therapy. This study was conducted on 100 non-duplicate *Acinetobacter* isolates that were collected from different patients admitted at Saint George Hospital-University Medical Center in Beirut. The isolates were identified using API 20NE strips, which contain the necessary agents to cover a panel of biochemical tests, and confirmed by PCR amplification of *bla*_OXA−51−like_. Activities of colistin, meropenem and imipenem against *Acinetobacter* isolates were determined by ETEST and microdilution methods, and interpreted according to the guidelines of the Clinical and Laboratory Standards Institute. In addition, PCR amplifications of the most common beta lactamases contributing to carbapenem resistance were performed. Tri locus PCR–typing was also performed to determine the international clonality of the isolates. Checkerboard, ETEST and time kill curves were then performed to determine the effect of the colistin-carbapenem combinations. The synergistic potential of the combination was then determined by calculating the Fractional Inhibitory Concentration Index (FICI), which is an index that indicates additivity, synergism, or antagonism between the antimicrobial agents. In this study, 84% of the isolates were resistant to meropenem, 78% to imipenem, and only one strain was resistant to colistin. 79% of the isolates harbored *bla*_OXA−23−like_ and pertained to the International Clone II. An additive effect for the colistin-carbapenem combination was observed using all three methods. The combination of colistin-meropenem showed better effects as compared to colistin-imipenem (*p* < 0.05). The colistin-meropenem and colistin-imipenem combinations also showed a decrease of 2.6 and 2.8-fold, respectively in the MIC of colistin (*p* < 0.001). Time kill assays additionally showed synergistic effects for a few isolates, and no bacterial re-growth was detected following a 24 h incubation. Our study showed that the combination of colistin with carbapenems could be a promising antimicrobial strategy in treating CRAB infections and potentially lowering colistin toxicity related to higher doses used in colistin monotherapy.

## Introduction

*Acinetobacter* spp. are organisms that could be found almost ubiquitously in nature. However, some species, especially *Acinetobacter baumannii* and its closely related species, have a great clinical significance in hospital environments since they are often associated with outbreaks and nosocomial infections (Towner, 2009; Howard et al., 2012). Multi-Drug Resistant (MDR) *Acinetobacter baumannii* is being increasingly implicated with infecting critically ill patients. The emergence of Carbapenem Resistant *Acinetobacter baumannii* (CRAB) strains and their detection in several regions across the world makes their treatment increasingly challenging (Towner, 2009; Howard et al., 2012).

A wide range of broad-spectrum antimicrobial agents have been used in the treatment of infections caused by MDR organisms. Of these agents, carbapenems are often resorted to due to their low toxicity and high efficacy (El-Herte et al., 2012). Nonetheless, the overuse and misuse of carbapenems led to an increase in resistance rates against this potent class of antimicrobial agents (El-Herte et al., 2012).

*A. baumannii* has several innate mechanisms of antibiotic resistance and a heightened ability to acquire resistance to numerous antimicrobial agents. This has led to the emergence of resistance among *A. baumannii* clinical isolates to a wide range of antimicrobial agents, including carbapenems (Howard et al., 2012). Oxacillinases (OXAs) are the most common cause of carbapenem resistance among this species. The intrinsic OXA-51-like is a chromosomally encoded beta-lactamase present in all *A. baumannii* isolates. Although this enzyme by itself does not convey carbapenem resistance, the association of its gene with an insertion sequence drives its over expression and leads to carbapenem resistance (Evans and Amyes, 2014). However, the main cause of carbapenem resistance is the acquisition of other types of oxacillinases (Howard et al., 2012). OXA-23-like, OXA-24-like, OXA-58-like, as well as the recently discovered OXA-143-like and OXA-235-like, are globally associated with the emergence of CRAB (Al Atrouni et al., 2016). Among these OXA families, OXA-23-like was found to be the most prevalent enzyme associated with CRAB infections in Lebanon. OXA-24-like and OXA-58-like have also been reported in this country, but at lower rates (Zarrilli et al., 2008; Al Atrouni et al., 2016). Similar to what is being reported all around the world, CRAB rates are being increasingly reported in Lebanon (Chamoun et al., 2016; Schultz et al., 2016). One Lebanese study showed an increase in the incidence of CRAB isolates from 50.8% in 2011 to 76.5% in 2015 (Al Atrouni et al., 2016; Chamoun et al., 2016). Moreover, International Clone II (ICII) was shown to be the most disseminated clone in this country (Al Atrouni et al., 2016; Schultz et al., 2016).

The high carbapenem resistance rates pose serious therapeutic and infection control challenges, especially since they are associated with high mortality rates and an increase in hospital stay (Zilberberg et al., 2016). Moreover, the lack of effective antibiotics against CRAB isolates led to the re-use of colistin (Neonakis et al., 2011). Colistin (polymyxin E) is a bactericidal antimicrobial agent that was reported to have relatively high levels of toxicity (Bialvaei and Samadi Kafil, 2015). Clinically, two types of colistin are available: Colistin sulfate and colistimethate, which is an inactive form of the drug that is converted to colistin sulfate after hydrolysis (Lim et al., 2010; Bialvaei and Samadi Kafil, 2015; Gurjar, 2015). Though data regarding the pharmacodynamic and pharmacokinetic properties of colistin is scarce, one study from Saudi Arabia showed that 76.1% of the patients treated with high doses of colistimethate developed acute kidney injury (Gurjar, 2015; Omrani et al., 2015). Neurotoxicity during aerolized colistin therapy has also been reported among 2.7% of patients suffering from ventilator associated pneumonia (Abdellatif et al., 2016).

Colistin has shown good *in vitro* activity against gram negative bacilli, including *A. baumannii*. However, resistance to colistin is emerging all across the globe (Ahmed et al., 2016). Colistin resistance in *A. baumannii* has been traced back to the loss or modifications of the Lipopolysaccharide (LPS) molecule (Bialvaei and Samadi Kafil, 2015; Lim et al., 2015; Cheah et al., 2016). A major cause for modifications of the LPS was found to be mutations in the *pmr*CAB operon (Lim et al., 2015), which have been reported to emerge during colistin therapy (Ni et al., 2015). Moreover, high doses of colistin were found to contribute to the emergence of colistin resistance in *Acinetobacter* spp., as well as to the emergence of heteroresistant *Acinetobacter* spp. isolates (Cheah et al., 2016). This, as well as the toxicity of this antimicrobial agent, led to the exploration of the possibility of using colistin in combination with other antimicrobial agents (Batirel et al., 2014). However, no standardized method for the *in vitro* evaluation of combination therapies was established by the CLSI (Tan et al., 2011). Nevertheless, studies have demonstrated a synergistic effect for the combinations of colistin-rifampin and colistin-vancomycin against MDR *A. baumannii* (Ahmed et al., 2016). Interestingly, colistin, vancomycin and rifampin are all antibiotics with relatively high levels of toxicity, whereas carbapenems have low levels of toxicity (Batirel et al., 2014). Nevertheless, few studies have addressed the therapeutic potential of the combination between colistin and carbapenems against CRAB isolates (Daoud et al., 2013). The aim of this study is to characterize *Acinetobacter* spp. isolates obtained from a Lebanese hospital and evaluate the *in vitro* effect of the colistin–carbapenem combination against these isolates using three different techniques.

## Materials and methods

### Study design and clinical isolates

This study was carried out at the Saint Georges Hospital-University Medical Center (SGH-UMC). It is a 400-bed tertiary care, educational, medical center located in central Beirut, Lebanon, that attends to around 22,000 admitted patients per year. A total of 100, non-duplicate consecutive isolates were collected from various clinical specimens recovered from 100 patients admitted to this hospital from June 2013 to June 2014. Only one isolate was collected from each patient, regardless of the site of infection it has been recovered from. All the isolates were stored at −80°C in Luria Bertani broth (LB) supplemented with 20% glycerol, and cultured on Mac Conckey agar (Oxoid) prior to testing. An IRB approval (IRB/0/037) was granted from the research committee at SGH-UMC. No written consent from the patients was taken since no interventions were performed.

### Identification and antimicrobial susceptibility testing

Conventional biochemical identification was performed using API 20NE strips (bioMérieux, Marcy l'Etoile, France) according to the manufacturer's instructions. The isolates were identified according to the databases provided by the manufacturers, based on the results of the panel of biochemical tests contained within the strips. Confirmation of the identification of *A. baumannii* was performed through the PCR amplification of *bla*_OXA−51−like_ (Le Minh et al., 2015). Susceptibility to different classes of antimicrobial agents was determined by the disc diffusion method (Clinical Laboratory and Standards Institute, 2015). In addition, Minimum Inhibitory Concentrations (MICs) of colistin, meropenem, and imipenem were performed by Etest MIC strips (BD, France, and Liofilchem®) and the broth microdilution methods (Clinical Laboratory and Standards Institute, 2015). Cutoff values were ≤ 2 μg/ ml≥ 4 μg/ ml for colistin and ≤ 2 μg/ ml≥ 8 μg/ ml for meropenem and imipenem (Clinical Laboratory and Standards Institute, 2015). The concentration ranges for the *E*-test were 0.016–256 μg/ ml for colistin and 0.002–32 μg/ ml for the carbapenems (BD, France, and Liofilchem®). The FDA tigecycline breakpoints for *Enterobacteriacaea* were applied to *Acinetobacter* spp. due to lack of breakpoint criteria in the CLSI guidelines (Stein and Babinchak, 2013).

### Polymerase chain reactions

DNA extraction was performed for all the isolates as described by Zhu et al. (1993). The DNA extracts were preserved at −20°C until used. *bla*_IMP_, *bla*_VIM_, *bla*_NDM_, *bla*_OXA−23−like_, *bla*_*OXA*−24−*like*_, *bla*_OXA−48_, *bla*_*OXA*−51−*like*_, *bla*_OXA−58−like_, *bla*_GES_, *bla*_KPC_, were tested for by Simplex PCR using the primers listed in Table 1 (Moubareck et al., 2009; Dallenne et al., 2010; Ardebili et al., 2014; Rafei et al., 2014). Positive and negative controls for the tested genes were provided from previous studies performed in the lab (Moubareck et al., 2009; Hammoudi et al., 2015).

**Table 1:** **primers used for PCR amplification with their different amplicon size**.

**Beta-lactamases**	**Bla gene**	**Primer direction**	**Sequence (5'–3')**	**Size (bp)**
**CLASS A**				
	GES	GES F	ATGCGCTTCATTCACGCAC	863
		GES R	CTATTTGTCCGTGCTCAGGA	
	KPC	KPC F	ATGTCACTGTATCGCCGTCT	881
		KPC R	TTACTGCCCGTTGACGCCCA	
**CLASS B**				
	IMP	IMP F	CGGCC (G=T) CAGGAG (A=C) G (G=T) CTTT	484
		IMP R	AACCAGTTTTGC(C=T) TTAC(C=T) AT	
	VIM	VIM F	ATTCCGGTCGG(A=G)GAGGTCCG	601
		VIM R	TGTGCTKGAGCAAKTCYAGACCG	
	NDM	NDM F	GGGCCGTATGAGTGATTGC	825
		NDM R	GAAGCTGAGCACCGCATTAG	
**CLASS D**				
	OXA-23-like	OXA-23-like F	ATGAATAAATATTTTACTTG	821
		OXA-23-like R	TTAAATAATATTCAGCTGTT	
	OXA-24-like	OXA-24-like F	ATACTTCCTATATTCAGCAT	809
		OXA-24-like R	GATTCCAAGATTTCTAGCG	
	OXA-48	OXA48 F	GCTTGATCGCCCTCGATT	281
		OXA48 R	GATTTGCTCCGTGGCCGAAA	
	OXA-51-like	OXA51-like F	TAATGCTTTGATCGGCCTTG	353
		OXA51-like R	TGGATTGCACTTCATCTTGG	
	OXA-58-like	OXA58-Like F	ATGAAATTATTAAAAATATTGAGT	840
		OXA58-like R	ATAAATAATGAAAAACACCCAA	

*R, reverse primer; F, forward primer.

### Clonal lineage

Tri-locus PCR typing was performed for the isolates that were confirmed as *A. baumannii* through the amplification of *bla*_OXA−51−like_ after biochemical testing by API 20NE strips (bioMérieux, Marcy l'Etoile, France), in order to determine the clonal relatedness of the *A. baumannii* isolates (Turton et al., 2007). This method consisted of performing two multiplex PCRs targeting different alleles of *ompA, csuE* and *bla*_OXA51−like_. The different amplification patterns were assigned to an international clone according to the patterns summarized by Karah et al. (2012).

### The checkerboard technique

The checkerboard technique was performed in triplicate using the combinations of colistin-meropenem and colistin-imipenem for all the 100 isolates obtained as previously described (Daoud et al., 2013). Concentration ranges of 32xMIC to 1/32xMIC for colistin and 8xMIC to 1/8xMIC for carbapenems were prepared in 96-well Microtiter plates (Thermo Scientific™). The concentration ranges were prepared in separate plates and then joined into a single plate so as to have different combinations of the antibiotics in each well. The bacterial inoculum was adjusted to 5 × 10^5^cfu/ml and distributed in all the wells. Two wells were reserved for positive and negative controls, respectively, in each plate. After incubation at 37°C for 24 h, the Fractional Inhibitory Concentration Index (FICI) was calculated using the formula “FICA + FICB = FICI” where “FICA” is the MIC of the drug A in combination/ MIC of the drug A alone; and “FICB” is the MIC of the drug B in combination/ MIC of the drug B alone (Daoud et al., 2013). The sum of FICI was then interpreted as follows: synergy if ∑FICI ≤ 0.5, additive effect if 0.5 < ∑FIC ≤ 2, indifference if 2 < ∑FIC ≤ 4, and antagonism if ∑FIC > 4 (Pillaii et al., 2005).

### Perpendicular *E*-test technique

**Figure 1:**
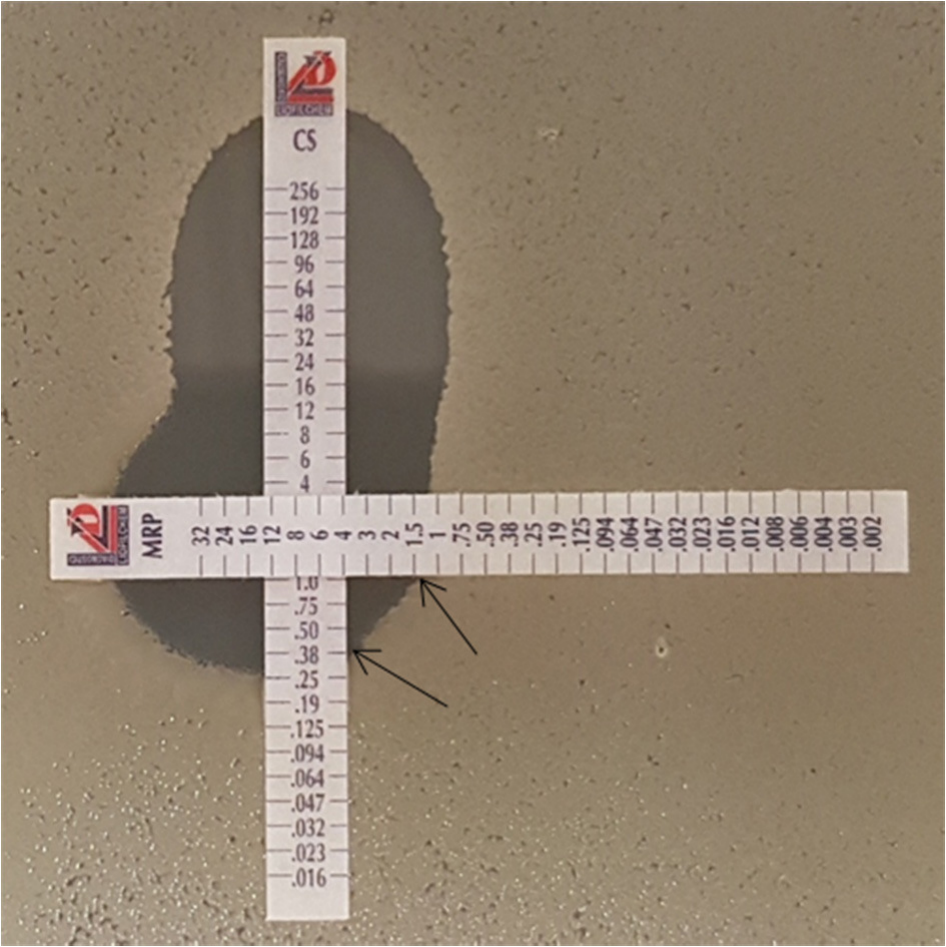
**Colistin-meropenem combination using the Etest (90°)**. Etest strip of colistin with either imipenem or meropenem was placed at 90°crossing at the MIC of the each drug as determined with previous test results. The black arrows indicate the antibiotic concentrations in combination. In this Figure, the MIC of meropenem was determined to be 1.5 μg/ml, and that of colistin in combination was 0.38 μg/ml. CS, colistin; MRP, meropenem.

Perpendicular *E*-tests were carried out in triplicate for 38 representative isolates. In this test, *E*-test strips containing colistin were placed perpendicularly with *E*-test strips containing either imipenem or meropenem over a lawn of the bacterial isolate on Mueller Hinton Agar plates (Figure 1). The plates were then incubated at 37°C for 18 h (Doern, 2014). The ∑FICI was then calculated and interpreted as described in the previous section (Pillaii et al., 2005).

### Time kill curve assay

Time-kill assays were performed in triplicate for 21 representative isolates, as described in the CLSI guidelines (2015). Briefly, concentration ranges of 16, 8, 4, 2, 1, 0.5, 0.25, and 0.125xMIC were prepared in Mueller Hinton Broth (Becton Dickinson, USA) for colistin, imipenem and meropenem alone, and in combination (colistin-imipenem and colistin-meropenem). A 5 x × 10^5^cfu/ml inoculum of the tested organism was also prepared and 1,000 μl were used to inoculate the 49 mL of the correspondent broth (containing the antibiotics alone, and in combination). The suspensions were then incubated at 37°C for 8 h with shaking at 200 rpm. An antibiotic-free growth control was also included. At predetermined time points (0, 1, 2, 3, 4, 5, 6, 7, and 8 h), 1,000 μl samples were aseptically acquired for Optical Density (OD) measurements at 580 nm. 100 μl samples were also obtained at these time points, serially diluted, and spread on Mueller Hinton agar plates in order to determine the colony forming units per mL (cfu/mL). Time kill curves were then constructed as a function of time and the results were represented as a difference in log10 between the cfu/mL at 0 and 8 h. A decrease of ≥ 3 Log10, as compared to the initial OD or cfu/ml, was indicative of a bactericidal effect (Doern, 2014). Synergistic effects were determined by a decrease of ≥ 2 Log10 in OD or cfu/ml when comparing the antibiotics in combination to the most active drug at that time point, while an increase of > 2 Log10 was considered as antagonism (Petersen et al., 2006; Doern, 2014). Additivity/indifference were interpreted as any other outcome that does not meet the criteria for either synergy or antagonism (Petersen et al., 2006).

### Statistical analysis

Normal distribution of the data was tested for using the Kolmogorov-Smirnov test. The data was found to be non-normally distributed and therefore the Wilcoxom and Mann-Whitney tests were carried out for statistical comparisons. *P*-values of less than 0.05 were considered as significant.

## Results

### Bacterial isolates and susceptibility testing

In this study, 95% of the isolates were identified as pertaining to the *Acinetobacter baumannii-calcoaceticus* complex, 3% as *A. haemolyticus*, 1% as *A. radioresistens*/*A. lwoffii*, and 1% as *A. junii*/*A. johnsonii* by the API 20NE strips (bioMérieux, Marcy l'Etoile, France). Forty percent of the isolates were recovered from patients from the Intensive Care Unit (ICU). Thirty eight percent of the patients were females with a median age of 70 and 62% of the patients were male with a median age of 72.5. As for the site of infection, 62% of the isolates were collected from the respiratory tract, 21% from pus, 5% from blood, and the rest of the isolates were obtained from other sources.

**Figure 2:**
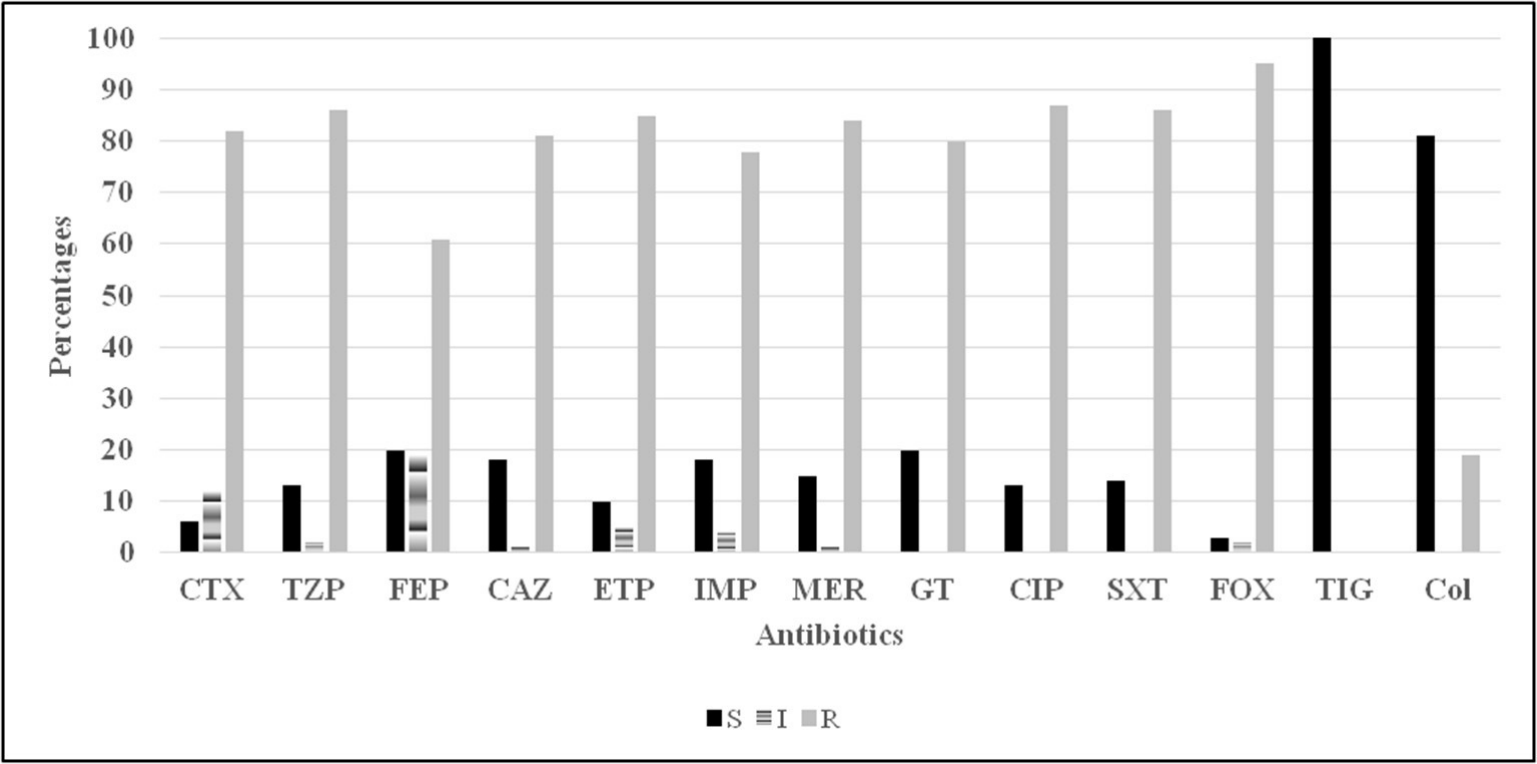
**Antibiotic susceptibility (disc diffusion method) profiles for 100 *Acinetobacter* spp. isolates**. CTX, cefotaxime; TZP, tazocillin; FEP, cefepime; CAZ, ceftazidime; ETP, ertapenem; Imp, imipenem; MER, meropenem; GT, gentamicin; CIP, ciprofloxacin; SXT, trimethoprim sulfamethoxazole; FOX, cefoxitin; TIG, tigecycline; Col, colistin.

In terms of the susceptibility patterns determined by the disc diffusion method, 84% of the isolates were resistant to meropenem and 78% were resistant to imipenem. 88% of the isolates were classified as MDR since they showed resistance to at least three categories of antimicrobial agents. Although the disc diffusion method showed a 19% resistance to colistin (Figure 2), the microdilution method showed that only one isolate was resistant to colistin. This difference in the rate of susceptibility between the two methods could be associated to the limited diffusion of colistin on solid agar (Van Der Heijden et al., 2007). Therefore, for the purposes of this study, the results of the microdilution method were considered for colistin resistance. The MIC_90_ and MIC_50_ of imipenem, meropenem, and colistin are shown in Table 2.

**Table 2:** **MIC50 and MIC90 of carbapenems and colistin**.

**Antibiotics**	**MIC 50(μg/ml)**	**MIC 90(μg/ml)**
Imipenem	8	32
Meropenem	16	32
Colistin	0.25	1

### Carbapenem resistance genes and international clonality

PCR amplification of the common carbapenemase genes showed that, *bla*_OXA−23−like_ was present in 79% and *bla*_OXA−24−like_ in 3% of the isolates. One isolate co-harbored *bla*_OXA−23−like_ and *bla*_OXA−24−like_. The chromosomal *bla*_OXA−51−like_ was detected in 99% of the isolates. The rest of the genes tested for were not detected in any of the isolates.

In terms of international clonality, 82 (86%) of the *A. baumannii* isolates pertained to international clone II (group 1), 6 (6.3%) to group 4, 3 (3.1%) to group 14, 1 (1.05%) to group 10, and 1 (1.05%) to group 8. Two (2.1%) isolates did not pertain to any group.

### *In vitro* combination effects

No synergistic effects were detected while combining colistin with the carbapenems using the checkerboard assay. However, additive effects were detected in both combinations where the Ẍ∑FICI of colistin combined with imipenem was 1.169 ± 0.354 and that of colistin with meropenem was 1.109 ± 0.337. The combination of colistin with meropenem showed a better additive effect than the colistin-imipenem combination (*p* < 0.05). The colistin-meropenem combination showed a better additive effect in meropenem resistant isolates when compared to meropenem susceptible isolates (*p* < 0.05) (Table 3). The combinations of colistin-imipenem and colistin- meropenem resulted in a decrease of 2.8- and 2.6-folds in the MIC of colistin, respectively. The additivity of the combination of carbapenem-colistin was also evident for the colistin resistant isolate 75.

**Table 3:** **Combinations against carbapenem-resistant and susceptible *Acinetobacter* spp**.

**Comb**	**Susceptible**			**Resistant**			**Impact**	***p*-value**
	**Nb**	**Mean**	**SD**	**Nb**	**Mean**	**SD**		
Col+Imp	21	1.233	0.387	65	1.138	0.37	Additive	**0.318**
Col+Mer	18	1.251	0.307	78	1.07	0.339	Additive	**0.035**
*p*-value	0.938			0.14				

*Col, colistin; Imp, imipenem; Mer, meropenem; Comb, combination; Nb, number of isolates. Non-bold p-values represent the significance between both combinations among resistant and susceptible isolates, respectively. The bold p-values represent the significance of colistin-imipenem and colistin meropenem combination between the susceptible and resistant isolates, respectively*.

Perpendicular *E*-tests also showed additive effects of the combinations where the Ẍ∑FICI of colistin combined with imipenem was 1.437± 0.41 and that of colistin with meropenem was 1.143 ± 0.50. However, no significant difference between the two combinations was found (*p* > 0.05). The combinations of colistin-imipenem and colistin-meropenem resulted in a decrease of 2.19- and 1.97-folds in the MIC of colistin, respectively.

Time kill curves showed that there was no bactericidal activity detected for all three antibiotics, where all the isolates showed an increase between 0.112 and 2.194 in cfu/ml from time 0 to 8 h. However, the cfu/mL values obtained were generally very low (Table 4). A significant bactericidal effect of colistin-imipenem when compared to colistin-meropenem (*p* < 0.05) was determined at 0.5XMIC. Moreover, no bacterial re-growth was detected at the different concentrations of colistin and colistin-carbapenem combinations. It is important to note that, due to limitations that were faced during the experiment, only 13 out of the 21 isolates were tested for at 16XMIC, which could have resulted in obtaining rather high values at this concentration as compared to the other concentrations.

**Table 4:** **ΔLog10 values of the cfu/ml of the *Acinetobacter* isolates obtained by the time kill curve assays after incubation, as compared to the initial inoculum**.

**Antibiotics**	**Bactericidal effect of colistin and carbapenem alone and in combination (time kill curve)**							
	**16xMIC**	**8xMIC**	**4xMIC**	**2xMIC**	**1xMIC**	**0.5xMIC**	**0.25xMIC**	**0.125xMIC**
	**ΔLog10**							
Col	0.429	0.155	0.214	0.441	1.18	1.368	1.621	1.826
Imp	0.325	0.139	0.137	0.121	0.218	1.12	1.914	2.174
Mer	0.413	0.131	0.116	0.282	1.12	1.922	2.054	2.194
Col-Imp	0.437	0.196	0.115	0.066	0.096	0.349	0.915	1.828
Col-Mer	0.403	0.178	0.098	0.195	0.106	0.815	1.06	1.802
*p*-value	0.528	0.698	0.679	0.103	0.831	0.018	0.112	0.731

*Col, colistin; Imp, Imipenem; Mer, meropenem; Col-Imp, colistin imipenem combination, Col-Mer, Colistin meropenem combination. A ΔLog10 value of ≥ 3 Log10 in cfu/ml determines a bactericidal activity*.

**Figure 3:**
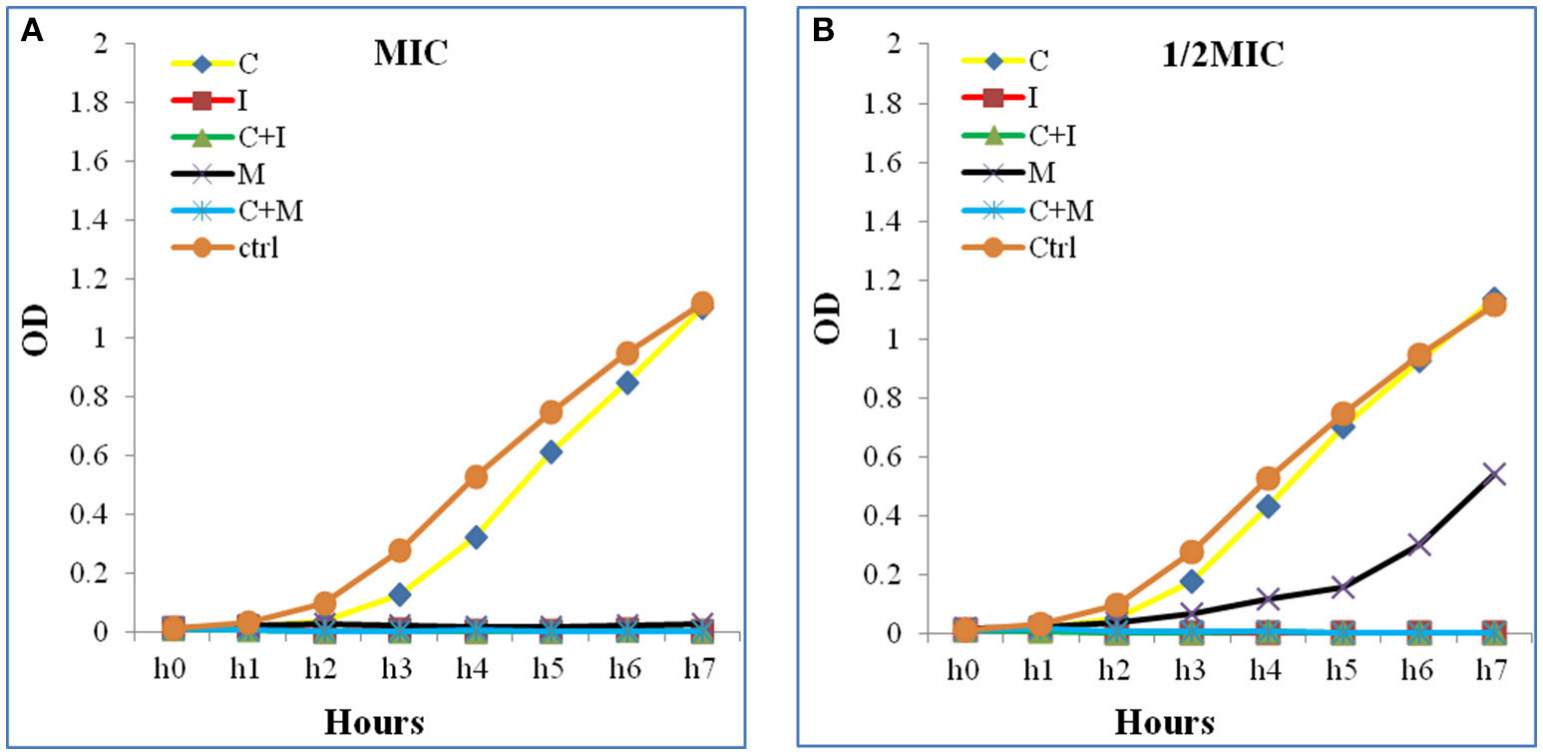
**Time kill curve assay chart showing the synergistic effect of a representative strain (A)** at 1 × 1MIC and **(B)** at 0.5 × 0.5 MIC. A significant decrease in the bacterial growth in both combinations (C+M and C+I) when compared to Colistin alone at 1 × 1 MIC and 0.5 × 0.5 MIC were detected. The Optical density (OD) is represented as a function of time. C, colistin; I, imipenem; C+I, colistin-imipenem; M, meropenem; C+M, colistin-meropenem; Ctrl, control.

Additive effects of the colistin-carbapenem combinations were observed for all the tested isolates (Table 5). Colistin-imipenem combinations showed better additivity than colistin-meropenem at 0.5 × 0.5MIC and 0.25 × 0.25MIC (*p* < 0.05). Synergistic effects for both combinations was detected in isolates 3, 13, 24, and 32 at 2 × 2MIC, 1 × 1MIC, 0.5 × 0.5MIC, and 0.25 × 0.25MIC, respectively (Figure 3, Table 6) by the time kill assays. No antagonistic effect was detected in this study and only isolate number 71 showed an indifferent effect using the checkerboard technique, but showed additive effects using the other techniques.

**Table 5:** **Synergistic effect of the combination of antimicrobial agents against *Acinetobacter* isolates**.

**Comb**	**Potential effect of time kill curve**							
	**16 × 16MIC**	**8 × 8MIC**	**4 × 4MIC**	**2 × 2MIC**	**1 × 1MIC**	**0.5 × 0.5MIC**	**0.25 × 0.25MIC**	**0.125 × 0.125MIC**
	**ΔLog10**							
Col-Imp	0.001	0.031	−0.11	−0.404	−1.077	−1.059	−0.732	−0.024
Col-Mer	−0.006	0.05	−0.119	−0.274	−0.937	−0.553	−0.554	−0.014
*p*-value	0.648	0.587	0.735	0.08	0.233	**0.012**	**0.031**	0.879

*Comb, combination; Col-Imp, colistin-imipenem combination; Col-Mer, colistin-meropenem combination. A decrease of ≥ 2 Log10 determines a synergistic effect. An increase of > 2 Log10 determines an antagonism. Additivity/indifference is determined for any outcome that does not fit with either synergistic or antagonism effect. Bold values indicates significant difference between combination*.

**Table 6:** **Comparative table of the three methods used**.

**Isolates**	**Comb**.	**Check**.	**Etest**	**Time kill (MICxMIC)**							
	**Drug A+B**	**FICI**	**FICI**	**16 × 16**	**8 × 8**	**4 × 4**	**2 × 2**	**1 × 1**	**0.5 × 0.5**	**0.25 × 0.25**	**0.125 × 0.125**
**(Δ Log 10)**											
3	C+I	1.20	1.16		0.60	−0.34	−0.84	−0.60	**−3.61**	−0.68	0.00
	C+M	1.05	1.60		0.74	−0.74	0.08	−0.60	−1.38	−0.06	0.00
4	C+I	1.04	2.00	0.01	0.05	0.02	−0.10	−0.01	−0.08	−1.37	
	C+M	1.04	2.00	−0.09	−0.23	−0.26	−0.33	−0.24	−0.26	−1.51	
8	C+I	1.41	2.00	−0.01	−0.01	−0.05	0.00	0.01	−0.02	−1.50	
	C+M	1.49	2.00	−0.01	−0.02	−0.03	−0.04	0.02	−0.02	−1.58	
13	C+I	1.41	1.60	0.14	0.05	−0.12	−0.42	**−2.65**	**−2.72**	**−2.71**	
	C+M	1.09	1.60	0.00	0.11	−0.11	−0.13	−0.37	**−2.08**	**−2.16**	
21	C+I	1.23	1.50	−0.01	0.00	−0.04	−0.03	−0.03	−0.02	−0.04	
	C+M	1.23	2.00	0.02	−0.01	0.00	−0.03	−0.03	−0.02	−0.02	
24	C+I	1.05	1.25		0.00	−0.34	−0.20	**−2.86**	−0.22	−0.05	−0.01
	C+M	0.98	1.75		0.40	−0.34	0.00	**−3.20**	−0.14	0.00	0.00
30	C+I	1.83	1.00		0.03	−0.02	−0.04	−0.02	−0.03	0.01	−0.34
	C+M	1.72	1.75		0.00	−0.03	−0.01	−0.02	−0.08	−0.14	0.02
32	C+I	1.05	2.00		0.14	−1.00	**−3.67**	**−3.68**	**−3.06**	−0.24	0.00
	C+M	0.94	1.75		0.00	−1.00	**−3.27**	**−3.68**	−0.13	0.03	0.01
34	C+I	0.99	1.25	−0.01	0.00	0.00	−1.25	−1.49	−1.54	−0.05	
	C+M	0.78	2.00	0.02	0.02	0.04	−1.21	−1.43	−0.16	0.04	
44	C+I	1.05	1.75	0.05	−0.05	−0.03	0.09	−1.33	−1.54	−0.30	
	C+M	0.61	1.16	−0.01	−0.10	−0.03	0.05	−1.33	−0.21	0.07	
48	C+I	1.42	1.40	0.03	0.05	0.02	0.06	0.44	0.00	−1.38	
	C+M	1.13	0.88	0.08	0.07	0.07	0.05	0.53	0.11	−1.43	
51	C+I	1.09	1.50		−0.01	−0.02	0.00	−1.07	−1.45	−1.59	−0.03
	C+M	0.81	2.00		0.01	0.03	0.03	−1.06	−1.42	−0.18	−0.02
63	C+I	1.33	1.40	−0.03	−0.03	−0.04	−0.03	−1.91	−1.46	−0.31	
	C+M	1.53	0.75	0.00	−0.01	0.03	−0.02	−1.33	−0.42	−0.14	
65	C+I	1.46	1.25		−0.04	−0.07	−0.07	−1.67	−1.74	−0.15	0.00
	C+M	1.05	1.25		−0.08	−0.12	−0.11	−1.71	−0.98	0.00	0.02
69	C+I	0.81	1.25	0.07	0.03	0.01	−0.09	−1.51	−1.63	−1.72	
	C+M	0.92	2.00	0.05	0.04	0.00	0.04	−1.38	−1.48	−1.38	
70	C+I	0.68	2.00		−0.05	−0.05	−0.06	−1.37	−1.30	−1.51	−0.03
	C+M	0.72	2.00		0.05	0.02	−0.04	−1.41	−1.48	−1.55	−0.06
71	C+I	2.54	2.00	−0.07	0.08	−0.05	0.09	−0.06	0.05	−0.04	
	C+M	2.09	2.00	0.00	−0.14	−0.11	0.00	−0.05	−0.03	−0.10	
73	C+I	0.67	1.25	−0.14	−0.07	−0.01	−0.66	−1.39	−1.50	−1.58	
	C+M	0.66	1.08	−0.12	−0.11	0.05	−0.64	−1.35	−1.51	−1.57	
75	C+I	0.92	1.00	−0.10	−0.11	−0.12	−0.29	−0.94	−0.02	0.00	
	C+M	0.68	0.85	−0.09	−0.09	−0.11	−0.29	−0.91	0.01	0.05	
78	C+I	0.83	1.60	0.09	0.02	0.00	−0.07	−0.08	−0.04	−0.10	0.00
	C+M	0.93	1.00	0.05	0.06	−0.02	−0.07	−0.07	−0.04	−0.10	
93	C+I	0.70	1.25		0.00	−0.07	−0.93	−0.40	−0.32	−0.07	0.21
		0.76	1.50		0.33	0.16	0.18	−0.07	0.10	0.11	−0.09

*C+I, Col-Imipenem; C+M, Col-meropenem. Comb, Combination; Check, Checkerboard. Synergistic effect is determined by ETEST and checkerboard whenever ∑FICI ≤ 0.5 was detected, and additivity was determined for the range 0.5< ∑FICI< 2. All the tested isolates showed additivity in both techniques. The values in bold represent a synergistic effect by time kill curve (< −2). At 2 × 2MIC: Synergistic rate: 4.7% for C+I and C+M respectively, at 1 × 1MIC: synergistic rate: 14.3% for C+I, and 9.5% for C+M, at 0.5 × 0.5MIC synergistic rate 14.3% for C+l, and 4.7% for C+M, at 0.25 × 0.25MIC synergistic rate of 4.7% for C+I and C+M, respectively*.

## Discussion

This study demonstrates the wide dissemination of MDR *Acinetobacter* spp. in SGH-UMC and highlights their dangerous potential in causing severe outbreaks. Our data also falls in line with local Lebanese data in terms of the predominance of IC II and *bla*_*OXA*−23−*like*_ (Al Atrouni et al., 2016).

The results of the combination experiments showed very high rates of additive effects when combining colistin with carbapenems using all three methods. This, however, does not fall in line with findings from another study conducted in Vietnam, where synergistic rates of 68 and 36% have been reported for colistin-meropenem and colistin-imipenem, respectively (Le Minh et al., 2015). The difference in both results determines how isolates pertaining to the same species are able to show diverse responses to antibiotics. This also falls in line with a previous study (Le Minh et al., 2015) that concluded that it is necessary to test *in vitro* combinations prior to *in vivo* use (Le Minh et al., 2015). The difference in results in different regions could be due to the difference in the genetic environments in which the strains are present, and the difference in exposure to antimicrobial agents. These factors could lead to the differential adaptation of the strains in different regions, and the subsequent difference in the interaction of the antimicrobial agents with these strains.

In this study, 99% of the isolates were susceptible to colistin as determined by microdilution method, advocating the use of this antimicrobial agent in monotherapy rather than in combination (Kara et al., 2015). Undoubtedly, the use of monotherapy could lower the chances of toxicity for the patient by avoiding the use of another antibiotic that could cause adverse side effects (Cetin et al., 2013). However, the use of colistin as monotherapy was shown to promote the emergence of heteroresistant *A. baumannii* isolates (Li et al., 2006; Lee et al., 2013) and the development of colistin resistance among other species such as *Pseudomonas aeruginosa*. Moreover, it was shown to promote the proliferation of species that have innate resistance to colistin, such as *Bulkhorderhia cepaciae*, (Cetin et al., 2013). Since these species are considered as highly potent pathogens, they have the potential of causing severe secondary infections following colistin treatment and leave physicians without any viable therapeutic options (Buford et al., 2016; Srinivasan et al., 2016).

Our results showed that combining colistin with carbapenems prevented bacterial re-growth and resulted in 2-fold decreases in colistin MICs. This has very promising implications in terms of using lower doses of colistin in therapy, and thus lowering its potential toxic effects. Moreover, this study showed a better additive effect of the colistin-meropenem combination as compared to the colistin-imipenem combination (*p* < 0.05). A previous study reported that the colistin-meropenem combination is more advantageous as compared to colistin-imipenem in the presence of *bla*_OXA−23−like_ (Daoud et al., 2013). However, in our study, no significant associations between *bla*_OXA−23−like_ and any particular combination were detected (*p* > 0.05). A probable explanation could be associated the higher affinity of meropenem, as compared to imipenem, to penicillin binding proteins in gram negative microorganisms (Le Minh et al., 2015).

With the exception of isolate 71, additivity was uniform while using all three different methods (Table 6). This holds true also for the colistin-resistant isolate (isolate 75), showing important implications for the combinations in the treatment of colistin-resistant strains. The additive rates were higher as detected by the checkerboard technique in comparison to the *E*-test (*p* < 0.05). A possible explanation could be the difference in characteristics between the liquid and the solid media that were used for these experiments (Luber et al., 2003). The checkerboard technique allows the determination of the optimal concentration of antibiotic capable of killing microorganisms at a determined incubation time (Doern, 2014). However, the time kill assay provides more accurate data regarding the effect of the combinations since the measurements are taken over time, and is able to detect bactericidal activities and bacterial re-growth (Doern, 2014). The higher accuracy of this assay as compared to the others could explain the detection of synergistic effects for some isolates that were not detected in other methods (Tables 4, 5). However, due to the labor-intensive nature of the experiment, it was not carried out for all the isolates included in this study. Nevertheless, our data showed a good agreement between the three methods that were used, as was reported by other studies as well (Petersen et al., 2006; Tan et al., 2011; Cetin et al., 2013). Based on that, the *E*-test method is recommended for use in routine clinical laboratories in case combinations are to be used in therapies since it is easy to perform and provides information regarding the effect of the combination in a rapid manner.

## Conclusion

In conclusion, a high rate of carbapenem resistance was detected among *A. baumannii* isolates obtained from SGH-UMC. ICII and *bla*_OXA−23−like_ were predominant among these isolates. Combining colistin with carbapenems showed high rates of additive effects using three different methods and resulted in a decrease in colistin MICs. These findings could be very promising in terms of using lesser concentrations of colistin in therapy while combining it with carbapenems and achieve good bacterial clearance rates. However, *in-vivo* experiments are needed before confirming that this combination could be used as a standard therapeutic option.

## Author contributions

MS, collected and identified the isolates, performed the susceptibility experiments, PCR experiments, combination techniques, statistical analysis. Was also involved in experiment design and data analysis and drafted the manuscript. ED, performed the tri-locus PCR experiments, clonality analysis, and revision of the manuscript. EA, data analysis. DS, study design and revision of the manuscript. ZD, was involved is study design, data analysis, revision of the manuscript and supervision of all the work performed at the lab.

### Conflict of interest statement

The authors declare that the research was conducted in the absence of any commercial or financial relationships that could be construed as a potential conflict of interest.
